# Glioblastoma Multiforme—Treating a Deadly Tumor with Both Strands of RNA

**DOI:** 10.1371/journal.pmed.0030031

**Published:** 2005-12-06

**Authors:** Robert J Weil

## Abstract

Weil discusses a promising new technique to deliver double-stranded RNA molecules to cancer cells, described in a new animal study in *PLoS Medicine*.

## Glioblastoma—The Clinical Challenge

Gliomas are the most common primary tumors of the brain, with an incidence of about 25,000 new cases per year in the United States [1]. At least half of all gliomas exhibit aggressive, malignant behavior. Glioblastoma multiforme (GBM), in particular, is clinically and pathologically malignant [1–3]. Patients with GBM have a poor prognosis, with a median survival of one year with aggressive therapy; fewer than 5% will survive five years [1,4,5]. In spite of its seemingly low incidence, mortality from GBM accounts for 3%–4% of all cancer deaths each year in the US [1].

The mainstays of treatment include surgical resection, radiation, and chemotherapy. Once adjuvant therapy is completed, gliomas generally recur at the surgical resection margin(s), and tend to be more aggressive than at initial presentation (Figure 1). At this stage in the course of disease, most therapy is palliative [1–5]. With the exception of a few early-stage clinical trials, current antiglioma therapies have not yet taken advantage of specific genetic abnormalities that lead to and sustain cancer. A new study by Alexander Levitzki and colleagues in this issue of *PLoS Medicine* presents promising preclinical results that appear to do just this, using a novel ligand-directed method to deliver double-stranded RNA molecules to cancer cells [6].

**Figure 1:**
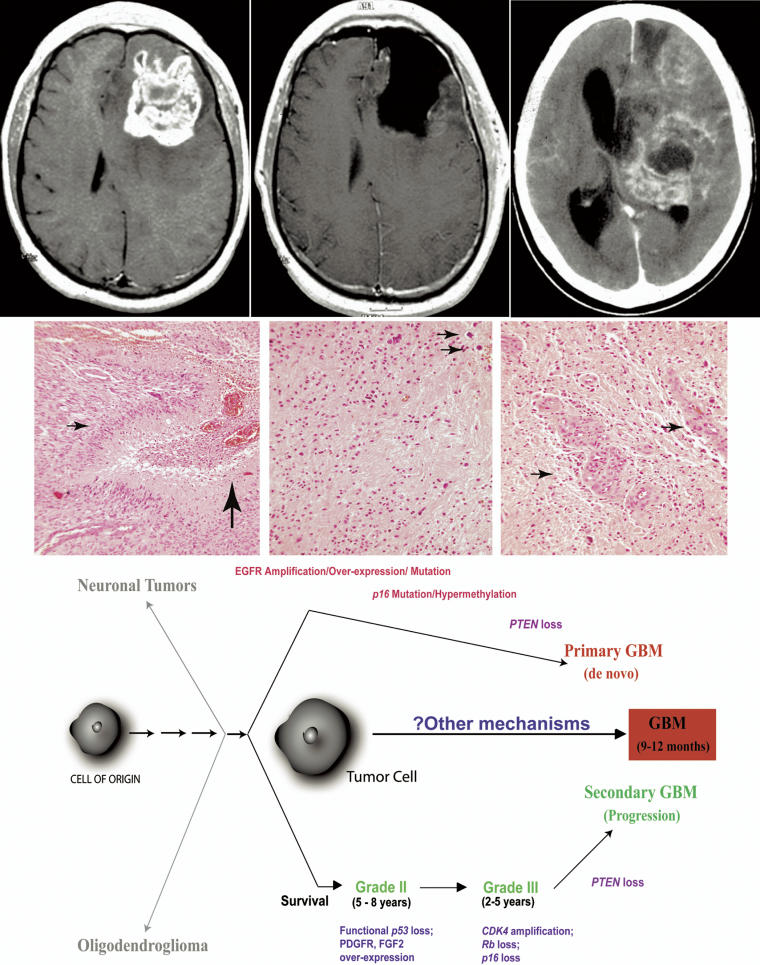
Representative Clinical, Pathological, and Molecular Genetic Features of Glioblastoma Multiforme The top panel shows an illustrative set of neuroimages from a patient with glioblastoma multiforme. On the left and in the center are gadolinium-enhanced T1-weighted axial magnetic resonance images from the day before and the day after resection of a large left frontal GBM. The patient was treated with postoperative radiation and chemotherapy. He presented again, 11 months after the first surgery, with stupor and a contrast-enhanced computed tomographic scan (right), which showed a massive and fatal recurrence. The middle panel shows histopathological examples of this patient's tumor. In the left image, there is evidence of hypercellularity, pseudopalisading necrosis (small arrow), and vascular proliferation with hemorrhage (large arrow). In the center image, there is hypercellularity with intermittent mitotic figures (small arrows), while in the image to the right, there are several areas with florid endothelial proliferation (small arrows). See also Table 1. All figures are 200× magnification. The bottom panel shows a schematic of current models of astrocytoma development and progression. The de novo pathway is located on top, and the secondary pathway is located on bottom. The principal genetic changes are noted for each pathway. Neuronal tumors and oligodendrogliomas (left top and bottom, respectively) appear to arise independently. Average survivals are noted for each astrocytoma type. While basic genetic features have been elaborated, they have been inconsistently found in GBMs, and as yet appear not to be consistently effective for targeted therapy. There remains a great deal unknown about the process by which these tumors progress to the most malignant state, whether the tumor is a de novo GBM or arises secondarily. Unknown steps from potential progenitor or pluripotent tumor cell(s) are indicated by small arrows.

**Table 1:**
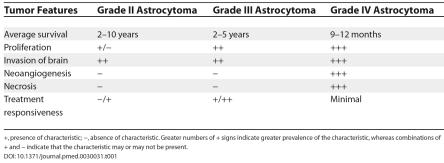
Histological Features and Prognosis in Patients with Glioma

+, presence of characteristic; −, absence of characteristic. Greater numbers of + signs indicate greater prevalence of the characteristic, whereas combinations of + and − indicate that the characteristic may or may not be present.

## Pathologic and Molecular Features

Gliomas are primary brain tumors that display pathological and ultrastructural features of glial cell differentiation. Primary brain tumors are classified on the basis of presumed line of neuroepithelial differentiation: astrocytic, oligodendroglial, and ependymal (Figure 1). Astrocytomas predominate, making up 80%–85% of all glial neoplasms, and will be the focus of this Perspective.

Grading is performed on a scale, from low to high, according to a tumor's histological features (Figure 1; Table 1). World Health Organization grade IV tumors, the GBMs, are aggressive, invasive, destructive malignancies, with increased mitotic activity, pronounced angiogenesis, necrosis, and proliferation rates two to five times higher than grade III tumors [2]. Roughly 50% of all GBMs are primary or de novo in origin, while the other half arises secondarily from lower-grade tumors [2], often after some years of latency [2]. Current models of gliomagenesis coincide with the two clinically recognized forms of GBM, de novo and progressive (Figure 1).

Most de novo GBMs do not have alterations in *TP53*; rather, nearly all carry EGF receptor *(EGFR)* gene amplifications, often combined with gene rearrangements that lead to a constitutively active, truncated receptor. By contrast, progression from a low-grade to a high-grade glioma often involves the serial accumulation of genetic alterations that inactivate tumor suppressor genes—such as *TP53, p16, RB, PTEN*—or activate oncogenes such as *MDM2, CDK4* and *CDK6* [2–4]. Functionally, gliomas seem to arise along two competing paths [3–5,7]. The first path is altered growth factor signaling—for example, activation of the EGFR-Ras-mitogen activated protein kinase, platelet-derived growth factor, or Akt pathways—which, both independently and through pathway crosstalk, lead to cell proliferation, cell cycle progression, and apoptosis inhibition (Figure 1). The second path is direct dys-regulation of cell cycle arrest, such as p16^ink4a^ control of Rb or p14^arf^ modulation of MDM2 and Tp53, among others.

## Current Diagnosis and Prognosis

At present, beyond the positive predictive value of increasing malignancy, as defined histopathologically (Table 1), survival of patients with GBM is predicated on clinical variables, including the patient's age and condition (Karnofsky performance score) at diagnosis, tumor location and extent of surgical resection, and administration of adjuvant radiotherapy and/or chemotherapy [1–5]. With respect to each modality—surgery, radiation, or chemotherapy—the survival advantage for each remains modest, on the order of a few months, with an average overall survival from the time of initial diagnosis of about 12 months. Therefore, therapies that promote a meaningful survival advantage, while promoting and enhancing quality of life, are urgently needed.

## Targeting Tumors with dsRNA

Because EGFR alterations are a common feature of many malignant tumors, including non-small-cell lung and colon cancers and malignant melanoma, among others, a variety of techniques have been designed to target the EGFR and its downstream agents, including antibodies, antisense RNAs, and a large number of small molecule inhibitors [7,8]. While many of these efforts have met with some success in other cancer types, none have had profound or lasting activity against GBM. Levitzki and colleagues have used a different strategy to target cells overexpressing EGFR: they use synthetic, double-stranded RNAs (dsRNAs), linked to EGF, to obtain selective and efficient killing of EGFR overexpressing malignant gliomas in vitro and in vivo in a mouse model [6].

dsRNA motifs are central to immune regulation, and dsRNA may play several roles in eukaryotic cells—blocking tolerance to tumor-associated self- and foreign antigens; activation of RNaseL and protein kinase R, which effect transcriptional and translational inhibition while simultaneously spurring interferon expression; induction of apoptosis; activation of small RNA-mediated interference; promotion of extracellular or paracrine effects through secretion of interferons and other cytokines (a “bystander effect’); and release of dsRNAs from infected cells, thereby activating antigen-presenting cells [9,10].

Given this panoply of potential effects, Shir et al. coupled dsRNA (a polyinosine-cytosine or poly IC construct) to EGF, and demonstrated that EGFR-targeted poly IC induced rapid and pronounced apoptosis of EGFR overexpressing cells, but not of cells expressing low EGFR, no EGFR, or mutated constitutively active EGFR, which cannot bind EGF. A variety of cytokines, including interferon-&alpha;, Gro-&alpha;, and interferon-induced protein-10/CXCL10—all of which have been shown to have antitumor or antiproliferative activity—were also expressed by the tumor cells. Importantly, these results were replicated in vivo, where dsRNA treatment led to survival of all animals with intracranial tumors for greater than 244 days. In addition, dsRNA treatment was equally applicable in vitro and in vivo for two other EGFR overexpressing cell lines, A431 (a cervical carcinoma) and MDA-MD-468 (a breast carcinoma), suggesting that this approach has potential for other tumor types that overexpress EGFR.

## Clinical Implications

While other treatments have had encouraging in vitro and in vivo debuts in animals, they have failed when translated to malignant gliomas in humans. Only clinical data will show whether the approach described above will be successful in human patients. However, there is reason for cautious optimism: based on recent advances in delivery of macromolecules to the brain, specifically by convection-enhanced delivery, pioneered by Edward Oldfield at the National Institutes of Health, it appears that ligand-guided delivery of dsRNA may hold significant clinical promise [11,12]. Convection-enhanced delivery permits selective delivery of heterogenous macromolecules to targeted-diseased regions within the brain both safely and efficiently, while minimizing or eliminating toxicities to the healthy brain or outside the central nervous system [11,12]. And since the system of Shir et al. can link dsRNAs to essentially any molecule, ligand-guided delivery might be broadly applicable to any cancer, and, quite likely, to benign disorders as well, so long as an endocytosed receptor is substantially overexpressed compared to normal cells. It appears to me that this is one method that should be fast-tracked to the clinic.

## Abbreviations

dsRNA: double-stranded RNA
GBM: glioblastoma multiforme
